# Origins of pandemics: Why ecological disruption is central to global health security and the imperative for proactive prevention

**DOI:** 10.1016/j.onehlt.2026.101598

**Published:** 2026-09-29

**Authors:** Shakeel Mahmood, Philip Naden, Subash Thapa, Peter Gibbs

**Affiliations:** a Rural Health Research Institute (RHRI), Charles Sturt University, Orange, NSW 2800, Australia; b Coonamble Aboriginal Health Service (CAHS), Coonamble, NSW 2829, Australia; c Regional Enterprise Development Institute (REDI.E), Dubbo, NSW 2830, Australia

**Keywords:** Pandemic origins, One Health, Planetary health, Zoonotic spillover, Indigenous ecological knowledge, Emerging infectious diseases

## Abstract

Pandemics are increasingly recognised as complex ecological and socio-economic phenomena rather than isolated biological events. The emergence of infectious diseases reflects dynamic interactions among humans, animals, and environmental systems, shaped by factors including biodiversity loss, land-use change, climate variability, urbanisation, and intensified human–wildlife contact. This viewpoint examines the evolving understanding of pandemic origins, highlighting the central role of ecological disruption and zoonotic spillover in shaping global health risks. The article further discusses the growing importance of One Health and planetary health frameworks in addressing pandemic prevention through integrated surveillance, cross-sectoral governance, ecosystem protection, and international collaboration. It also highlights the critical contribution of Indigenous ecological knowledge, which emphasises reciprocal relationships between humans and nature and provides valuable insights for biodiversity conservation, climate adaptation, and disease prevention. Strengthening global preparedness requires moving beyond reactive outbreak responses towards proactive strategies that address the ecological and societal drivers of pathogen emergence. Understanding pandemic origins is therefore not only a scientific priority but also a global governance imperative for protecting planetary and human health.

## 1 Introduction

Understanding the origins of pandemics has gained renewed importance in global health, extending beyond COVID-19 to broader patterns of emerging infectious diseases. Increasing evidence indicates that these diseases do not arise as isolated biological events but emerge from complex interactions among humans, animals, and environmental system [1], [2]. This viewpoint highlights the central role of ecological disruption, human behaviour, and socio-economic change in shaping the conditions that enable pathogen emergence and transmission.

Early ecological scholarship anticipated this paradigm. In 1999, planetary scientist Emeritus Professor M. R. Choudhury proposed that disruptions to ecological balance could contribute to the emergence and re-emergence of pathogenic microorganisms. His observation that “microbes can strike back when ecological balance is disturbed” reflects a concept increasingly supported by contemporary research, which identifies ecosystem integrity as a fundamental determinant of global health security [3], [4].

Similarly, Stephen S. Morse, Professor of Epidemiology highlighted in 2012 that most pandemics originate from animal reservoirs and are predominantly caused by viruses. He further emphasised that pathogen emergence is shaped by ecological, behavioural, and socio-economic changes that alter interactions between humans, animals, and the environment [4]. Andrew P. Dobson, Professor of Ecology (2020), identified tropical and subtropical forests as important reservoirs for emerging pathogens, highlighting how ecological degradation and environmental disruption can increase the risk of zoonotic spillover [5]. Peter Piot, Professor of Global Health (2023), has emphasised the importance of strengthening preparedness for future pandemics by addressing the ecological and systemic drivers that contribute to disease emergence [6]. More recently, Raina K. Plowright, Professor of Ecosystem (2024) highlighted that integrating ecological measures into pandemic prevention policies can reduce future pandemic risk by protecting and restoring ecosystems and addressing environmental and ecosystem-level drivers of pathogen spillover and zoonotic transmission [7].

Consistent with this viewpoint, *The Lancet* (2024) and *The Nature (*2026) allies with these observations, indicating that the pandemic possibly originated from natural processes, with SARS-CoV-2 being transmitted to humans via contact with wildlife. Debates over COVID-19 origins distract from collaborative efforts needed to strengthen global health, pandemic prevention, and preparedness [[8], [9]].

Human–animal interactions, pathogen emergence, and pandemic preparedness provide a foundation for an ecological and systems-oriented understanding of pandemic risk. However, this selection does not encompass the full range of determinants of pandemic emergence. A more comprehensive understanding should also consider socioeconomic drivers, food systems and food security, host and immune vulnerability, and pathogen adaptation across animal reservoirs and over time and geography, creating new genovariants [10].

Importantly, these processes are not independent of the broader systems in which they occur. Economic structures, trade networks, development pathways, institutional arrangements, governance systems, and cultural practices shape patterns of production and consumption, resource use, land-use change, and human–animal interactions [11]. Ecological disruption can also affect food systems, environmental contamination, nutritional status, and host vulnerability, while sustained pathogen transmission provides opportunities for evolutionary adaptation [12]. From this viewpoint, ecological disruption remains a central pathway to pandemic emergence, but it should be understood within the wider socioeconomic, institutional, cultural, biological, and environmental systems that produce and amplify these conditions [11].

Accordingly, the “origin” story should extend beyond the point at which a pathogen emerges to consider the upstream systems, policies, institutions, and knowledge practices that drive anthropogenic environmental change, alter ecosystems and human–animal relationships, influence food and health security, and create conditions conducive to pathogen emergence and adaptation [11].

Further contributing to this evidence base, recent research has indicated that zoonotic spillover remains the most strongly supported explanation for the emergence of SARS-CoV-2, while acknowledging that the precise origin remains unresolved due to persistent gaps in available evidence [9]. Collectively, these findings reinforce that understanding pandemic origins requires an integrated approach that considers ecological change, human–animal interactions, environmental governance, and global preparedness within a broader planetary health framework.

While this viewpoint emphasises ecological mechanisms, it recognizes that a comprehensive understanding of pandemic origins also requires consideration of socio-economic systems, food security, and pathogen adaptation across reservoirs. These complementary viewpoints help situate ecological disruption within the wider social, biological, and economic processes that shape pathogen emergence and transmission. This viewpoint examines ecological mechanisms, considers relevant governance frameworks, and highlights the contribution of Indigenous knowledge to pandemic prevention and preparedness.

## 2 Ecological drivers of zoonotic spillover

A substantial body of evidence indicates that most emerging infectious diseases arise through zoonotic spillover, whereby pathogens are transmitted from animals to humans. These events are increasingly recognised as consequences of complex interactions between ecological disruption, human activities, and changing environmental conditions. Key anthropogenic drivers include deforestation, biodiversity loss, land-use intensification, agricultural expansion, urbanisation, climate change, global trade, and increasing human–wildlife interactions. Together, these factors have contributed to the emergence of many pandemics over the past century and are closely associated with patterns of human population density and global biodiversity distribution [13].

Ecological disruption increases zoonotic risk through three interconnected mechanisms: direct spillover, by increasing human–animal contact; host vulnerability, through food insecurity, pollution and contamination that may increase host vulnerability; and pathogen adaptation, as environmental change and new host interactions create opportunities for genetic evolution and the emergence of new variants [14]. Pathogens adapt to new reservoirs through genetic changes and ecological pressures. Genetic changes can enhance pathogen fitness, while environmental disruption and increased cross-species contact facilitate spillover; continued transmission may drive adaptation to new hosts [15].

Deforestation, land-use change, and agricultural expansion can fragment habitats, reduce biodiversity, alter wildlife behaviour, and increase contact among wildlife, livestock, and humans, thereby creating conditions that may facilitate pathogen spillover [14]. These practices contrast with many Indigenous approaches to land and resource management, which emphasise relational responsibilities, ecological stewardship, seasonal knowledge, biodiversity protection, and sustainable use of resources [16]. By maintaining ecological connectivity, respecting species relationships, and regulating resource use, these approaches can reduce ecological disruption and zoonotic transmission risks. Understanding these differences is important because the way land and resources are managed can shape both ecosystem health and human–animal disease risk.

Global connectivity further amplifies pandemic risk by facilitating the rapid movement of pathogens across geographic boundaries, with highly connected urban centres acting as focal points for disease transmission and epidemic expansion [17]. Similarly, urbanisation has been identified as a significant biosecurity risk [18]. Urban environments are increasingly recognised as hotspots for emerging infectious diseases due to their high population density, extensive mobility networks, and frequent interactions among humans, animals, and modified ecosystems [19]. Environmental degradation further increases spillover risk by disrupting wildlife habitats and bringing animal reservoirs into closer proximity with human settlements, thereby creating conditions favourable for pathogen emergence [4].

Climate change represents an additional and growing driver of infectious disease risk by altering ecological conditions that influence pathogen survival, vector distribution, and transmission dynamics. Changes in temperature, humidity, and rainfall patterns can expand the geographic range of pathogens and vectors, exposing previously unaffected populations to emerging infectious threats [20]. Extreme heat events further intensify vulnerability by interacting with displacement, overcrowding, water scarcity, and inadequate energy infrastructure. These compounding factors increase the risks of heat-related illnesses and infectious diseases [21] and approximately 2.5 billion cases of human illness and 2.7 million deaths each year globally [22].Together, these interconnected ecological, climatic, and social processes increase the likelihood of pathogen emergence at the human–animal interface, highlighting the importance of a planetary health perspective.

Historical outbreaks provide compelling evidence that pandemic emergence follows recurring ecological patterns rather than isolated stochastic events. Over 60% of existing and 75% of emerging and re-emerging human diseases are zoonotic [23]. Most zoonotic disease events involve domestic animals, whereas wildlife are the primary reservoirs of emerging infectious diseases [24]. Additionally, over 36% of emerging and re-emerging zoonotic diseases are associated with food-producing animals. [24]. A 2022 report further noted that, between 1940 and 2004, nearly two-thirds of the 335 documented emerging infectious diseases (EIDs) were first detected through surveillance systems, with many originating from wildlife sources, emphasising the importance of early detection and ecological monitoring [25].

Influenza provides a particularly important illustration of this dynamic. Human pandemic and seasonal influenza viruses originated from enzootic influenza viruses circulating in wild waterfowl and shorebirds. Genetic evidence shows the 1918 influenza pandemic arose from an avian host switch, leading to later pandemics and seasonal influenza. Avian influenza viruses have crossed species barriers, infecting horses, pigs, dogs, seals, and other vertebrates, demonstrating ongoing pandemic potential [26]. Likewise, major outbreaks, including HIV/AIDS, SARS, MERS, Ebola virus disease, H1N1 influenza, avian influenza, Zika virus disease, measles, cholera, smallpox (eradicated in 1980), falciparum malaria, dengue and Nipah virus infection, demonstrate the persistent role of zoonotic transmission in shaping global health threats [27].

For example, ecological modification of pig farms in Malaysia during the 1990s facilitated Nipah virus transmission from fruit bats to pigs and subsequently to humans, with recurrent outbreaks continuing in Bangladesh and India [28]. Similarly, the identification of raccoon dog DNA as a potential intermediate host associated with SARS-CoV-2 highlights the continued importance of wildlife reservoirs and intermediary hosts in the emergence of novel zoonotic pathogens [25].

Recent outbreaks further demonstrate the ongoing vulnerability of global health systems in an increasingly interconnected world. Emerging events in 2026, including Ebola virus disease activity in Central Africa and cross-border clusters of hantavirus infections associated with international travel, underscore the continued threat posed by zoonotic pathogens [[29], [30]]. Similarly, the first detection of H5N1 avian influenza in mainland Australia in 2026 represents a notable geographic expansion of the virus, highlighting how ecological change, wildlife movement, including wildlife market and trade, and global connectivity continue to reshape patterns of infectious disease emergence [31].

Following the 2002 SARS outbreak, global attention to emerging coronaviruses declined; however, 18 years later, COVID-19 emerged as the deadliest respiratory pandemic since the 1918 Spanish influenza pandemic, which caused an estimated 50 million deaths [26]. These events highlight that addressing ecological drivers through restoration and ecosystem protection is essential for preventing zoonotic spillover and strengthening global health security.

## 3 Conceptual framework

As a viewpoint article, this paper does not require a conventional Methods section. Instead, it adopts three complementary viewpoints: Ecology, One Health and Planetary Health, and Indigenous Knowledge to examine the interconnected drivers of pandemic emergence and prevention.

Ecology provides the primary lens for understanding how biodiversity loss, land-use change, climate change, and ecosystem disruption alter human–animal interactions and increase zoonotic risk [32]. One Health and Planetary Health provide broader systems frameworks linking human, animal, environmental, and planetary health, while supporting integrated surveillance, cross-sectoral governance, prevention, and preparedness [33]. Indigenous Knowledge complements these viewpoints by emphasising reciprocal relationships with Country, environmental stewardship, place-based knowledge, and intergenerational responsibility [34].

These frameworks were selected because they collectively support an ecological, systems-oriented, and culturally informed understanding of pandemic risk. However, they do not provide an exhaustive account of pandemic origins. Socio-economic and political-economic systems, food systems and food security, immune vulnerability, and pathogen adaptation important complementary viewpoint but are beyond the primary focus of this viewpoint [11]. Recognising these limitations, the framework provides the conceptual basis for examining ecological drivers, governance responses, and Indigenous contributions to pandemic prevention and preparedness.

## 4 Global health responses: One Health and governance frameworks

In response to the growing complexity of pandemic risks, global health governance has increasingly embraced the One Health approach, recognising the interconnectedness of human, animal, and environmental health. Within a planetary health framework, it emphasises that pandemic prevention requires integrated surveillance systems, enhanced biosecurity, cross-sectoral collaboration, strengthened laboratory capacity, and coordinated governance mechanisms at the human–animal–environment interface [35].

The Quadripartite One Health Joint Plan of Action (2022–2026) provides a comprehensive framework for translating One Health principles into practice through six strategic action tracks, supporting coordinated efforts to address zoonotic diseases, antimicrobial resistance, ecosystem degradation, and other emerging health threats [36]. Additionally, experts underscored the One Health approach as an essential cross-cutting framework to confront complex global challenges, including zoonotic diseases and climate-related health risks, thereby directly supporting Sustainable Development Goals (SDGs) attainment [36]. Together, these developments underscore that pandemic prevention depends on sustained investment in integrated governance systems capable of addressing ecological and social determinants of disease emergence.

This evidence highlights the importance of transparency, data sharing, and international collaboration to strengthen outbreak investigation and preparedness. Within the broader concepts of planetary health and planetary defence, preparedness is increasingly viewed as a proactive and interconnected global responsibility, integrating scientific innovation, technological advancement, and international collaboration [37]. This evolving paradigm represents a shift from reactive responses to outbreaks towards anticipatory approaches focused on prevention, resilience, and planetary stewardship [37]. However, persistent challenges in data accessibility, surveillance capacity, and international information sharing continue to limit a comprehensive understanding of pathogen emergence pathways and constrain effective pandemic preparedness.

## 5 Indigenous knowledge and resilience

The growing recognition of Indigenous knowledge reflects a broader transformation in global environmental governance, where Indigenous knowledge systems are increasingly valued as complementary to scientific approaches for understanding complex ecosystem dynamics and long-term environmental change [38]. From an Indigenous Peoples' perspective, knowledge is not only informational but relational embedded in responsibilities to Country, stewardship obligations, and intergenerational continuity meaning that environmental governance is strengthened when Indigenous self-determination and data sovereignty are respected alongside scientific collaboration [39].

This cultural dimension is a missing link between ecological disruption and human behaviour and zoonotic risk, highlighting the distinctive value of Indigenous knowledge. How people relate to environments influences resource use, animal interactions, responses to change, and collective ecosystem management. Culture therefore helps shape the social norms, institutions, practices, and priorities through which ecological relationships are maintained, transformed, or disrupted [40].

Indigenous knowledge views humans as interconnected with ecosystems, emphasising relationships, responsibilities, and ecological interdependence. These relationships have shaped cultures, institutions, and social priorities oriented towards long-term stewardship, reciprocity, and responsibility to future generations. Such perspectives can provide an important counterpoint to development models that prioritise short-term resource extraction and economic gains over ecological integrity [40]. Modern industrial societies, and increasingly developing societies following similar development pathways, may also become culturally disconnected from their local environments. Environmental disconnection may weaken ecological awareness and contribute to behaviours and practices and increase habitat degradation, wildlife exploitation, human–animal interactions, and zoonotic spillover risks [41].

Indigenous ecological knowledge, developed through sustained relationships with land, water, and biodiversity over generations, offers context-specific insights into species behaviour, seasonal variability, and environmental thresholds that are often difficult to capture through conventional monitoring systems alone [42]. Intergenerational Indigenous knowledge supports understanding ecological change and consequences of disrupting relationships among people, animals, and ecosystems [43]. In this sense, Indigenous knowledge is not simply a repository of ecological observations; it also embodies cultural values, teachings, responsibilities, and reciprocal relationships with Country that can guide everyday practices, environmental stewardship, and resource management [40]. When integrated with contemporary scientific methods, these knowledge systems can enhance early warning capacities for ecological disruption, improve biodiversity conservation strategies, and strengthen community-based adaptation to climate and health risk [44].

Evidence from Indigenous-led conservation and One Health initiatives demonstrates the practical value of such integration. In northern Australia, Aboriginal fire management programs in Arnhem Land combine Indigenous fire knowledge with carbon accounting and ecological science, resulting in reduced emissions, improved biodiversity outcomes, enhance biosecurity in animal husbandry and strengthened Indigenous land governance [45]. In Aotearoa New Zealand, recognition of the Whanganui River as a legal entity through Te Awa Tupua reflects Māori relationships with waterways and provides a model of integrated environmental governance based on Indigenous values, ecosystem protection, and shared accountability [46]. In the Canadian Arctic, Inuit-led monitoring programs integrate Indigenous knowledge with scientific and satellite data to track wildlife migration, food security risks, and climate-sensitive zoonotic threats, demonstrating the relevance of Indigenous knowledge for One Health surveillance [47]. Similarly, Native American and Alaska Native ecological knowledge reflects a relational worldview in which humans, animals, and ecosystems are interconnected, closely aligning with One Health principles [48].

Comparable examples from South Asia further demonstrate the contribution of Indigenous ecological knowledge to One Health approaches. Indigenous communities in Bangladesh maintain holistic ecological practices centred on sustainable forest stewardship, biodiversity conservation, traditional agriculture, and medicinal plant use. These practices support ecosystem resilience, food security, zoonotic disease prevention, and climate adaptation [49]. Likewise, Indigenous and rural communities in Nepal apply ecological knowledge systems based on interconnected human–animal–environment relationships, sustainable resource management, and ethno-medicinal practices. Although Nepal has advanced One Health initiatives to address zoonotic diseases and environmental change, challenges remain in implementation, particularly in rural and resource-limited settings. Integrating Indigenous knowledge into surveillance, governance, and health strategies could strengthen ecosystem resilience, public health outcomes, and sustainable health systems [50].

Increasingly, international organisations recognise Indigenous knowledge as a critical resource for biodiversity conservation, climate adaptation, and pandemic preparedness. This recognition reflects the understanding that Indigenous stewardship grounded in reciprocal relationships with land and living systems can enhance ecosystem resilience, promote public health, and support sustainable environmental governance [49]. (Table 1). Evidence supporting ecological resilience and sustainable management justifies incorporating Indigenous knowledge and leadership into pandemic prevention and Planetary Health strategies [51]. Frameworks such as the United Nations Declaration on the Rights of Indigenous Peoples (UNDRIP) further provide an important foundation for inclusive and ethical governance by affirming Indigenous rights, self-determination, and participation in decisions affecting their lands, knowledge systems, and communities [52].

**Table 1:** The Indigenous worldview closely aligns with the principles of the One Health, which recognizes that human, animal, and environmental health are interconnected.

Indigenous ecological perspective	One Health principle
Humans are part of ecosystems	Human, animal, and environmental health are interconnected [53]
Protection of forests and biodiversity	Healthy ecosystems reduce disease emergence [54]
Traditional livestock and wildlife management	Supports prevention of zoonotic diseases, sustainable food systems and ecosystem health [53]
Sustainable agriculture	Promotes food and socio-economic security and environmental health [55]
Cultural burning and land stewardship	Improves ecosystem resilience and reduces environmental risks [56]
Community stewardship of natural resources	Encourages participatory and locally led One health governance [53]

Together, this evidence and rights-based foundation support strategic actions that move beyond recognising Indigenous knowledge to ensuring meaningful Indigenous participation, leadership, and decision-making in environmental and pandemic-prevention policies [57]**.** Recognising these rights is essential for developing more effective approaches to pandemic prevention, ecological protection, and planetary health governance (Fig. 1).

**Fig. 1:**
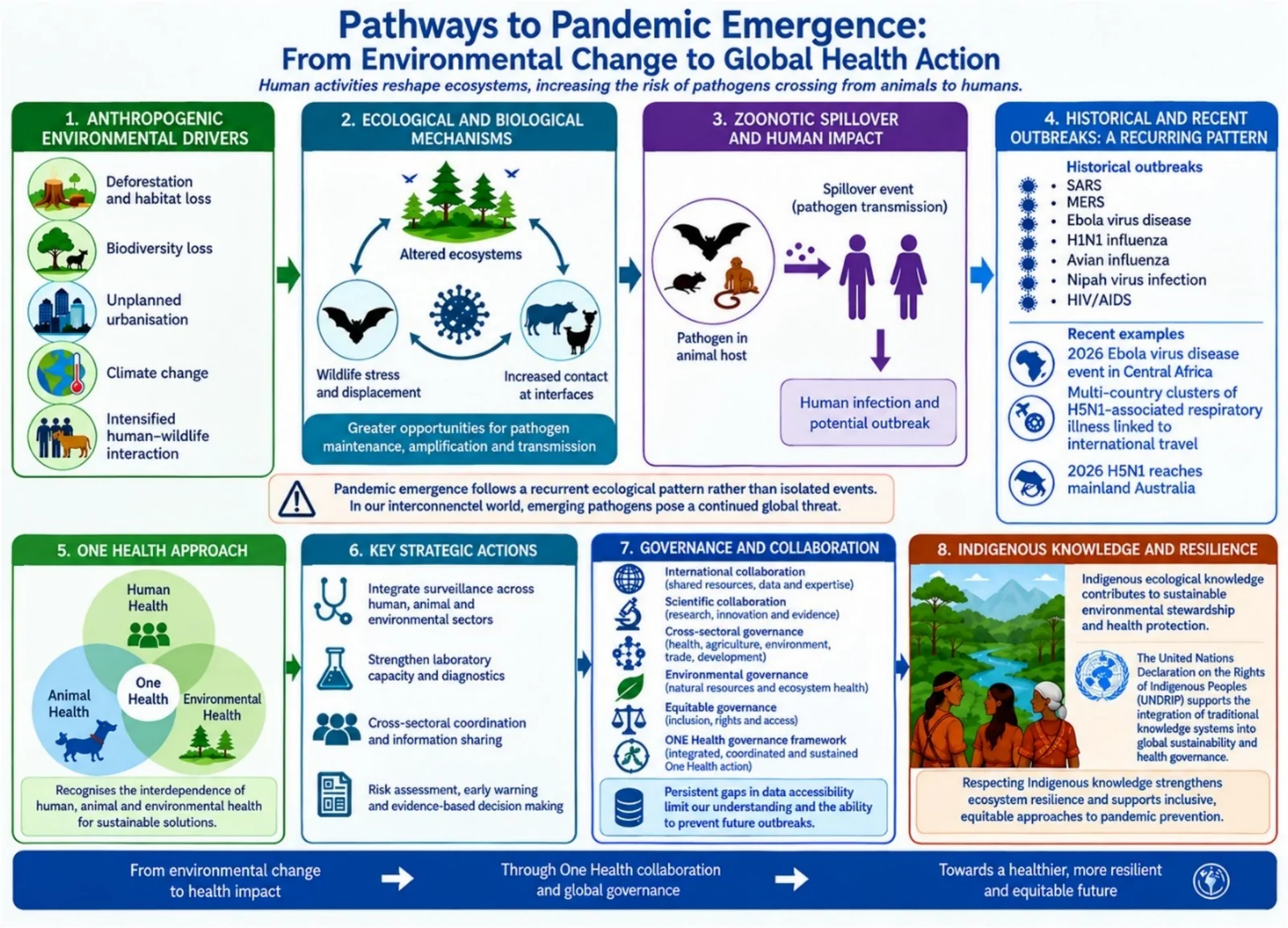
Pathways to Pandemic Emergence: From Environmental Change to Global Action.

## 6 Conclusion

The world has entered a new pandemic era, characterised by the increasing frequency of epidemics and pandemics, some of which may involve highly pathogenic infectious agents [26]. Pandemics should not be viewed solely as random biological events, but as outcomes of complex interactions among ecological, climatic, social, cultural, and socio-economic systems. Social and economic structures shape how societies use land, manage natural resources, produce and consume food, and interact with wildlife. These practices can contribute to deforestation, biodiversity loss, agricultural expansion, pollution, and other forms of ecological disruption that alter ecosystems and increase opportunities for zoonotic spillover [58].

Understanding pandemic origins requires examining upstream ecological, social, economic, food-security, and pathogen-adaptation processes preceding human infection. Ecological disruption can alter wildlife habitats and human–animal interfaces, increase opportunities for pathogen spillover, and create conditions that facilitate pathogen emergence and transmission [59]. The connection between a society and its local environment is therefore not merely an ecological fact; it is also a cultural achievement that shapes social priorities, governance structures, and the collective actions through which ecosystems are either protected or degrade ecosystem integrity [40]. Cultural values and relationships with the environment also influence these practices, shaping approaches to environmental management and stewardship [58]. Thus, the “origin” of a pandemic should not be conceptualised as a discrete event, time, or geographical location, but rather as a dynamic, multistage socio-ecological process arising from the interaction of socioeconomic, environmental, biological, cultural, and institutional pathways, including ecological disruption, host vulnerability, pathogen adaptation, and subsequent human transmission. [60]. Understanding these pathways requires examining how political economy, trade, resource extraction, institutions, and development shape land use and environmental change [14]. Future research should examine how structural forces and cultural relationships shape ecological integrity, human–animal interactions, and zoonotic emergence.

This article argues that pandemics arise from complex ecological and socio-economic drivers rather than isolated biological events, and asserts that effective prevention requires integrated approaches grounded in One Health, planetary health, and Indigenous ecological knowledge. It maintains that strengthening global preparedness demands proactive strategies that address the environmental and societal determinants of pathogen emergence, positioning pandemic-origin research as a critical scientific and governance priority for protecting planetary and human health.

The risk of future viral outbreaks remains high [3], [6], underscoring the need for a more proactive approach to pandemic prevention. One Health High-Level Expert Panel have similarly emphasised reducing zoonotic spillover risk at source rather than relying primarily on downstream detection and response [61]. This viewpoint therefore highlights the importance of addressing the upstream drivers of ecological disruption alongside strengthening surveillance, public health preparedness, international collaboration, and scientific capacity. It also emphasises the value of Indigenous knowledge systems, which offer longstanding approaches to environmental stewardship and relationships with Country that can complement One Health and planetary health approaches [62].

High-density farming systems, combined with weak surveillance and health systems, can increase opportunities for pathogen emergence and delay timely detection and response. Pandemic risk therefore arises from interactions between ecological disruption, human activities, and governance gaps, underscoring the need for an integrated One Health approach. Addressing these risks requires changes in human behaviour and practices that contribute to ecosystem degradation and disrupt human–animal–environment interactions. Such practices exacerbate environmental injustice and ecosystem degradation. Extending the ethical principle of “do no harm” beyond human health to encompass animals and ecosystems reinforces humanity’s responsibility for environmental stewardship. Sustainable land use, responsible agricultural practices, and effective environmental governance can help reduce opportunities for pathogen spillover and infectious disease emergence. Integrating One Health, planetary health, Indigenous knowledge systems, public health preparedness, and equitable governance is therefore essential to addressing the ecological, social, and cultural drivers of pandemics and strengthening sustainable global health security.

## CRediT authorship contribution statement

**Shakeel Mahmood:** Writing – review & editing, Writing – original draft, Supervision, Resources, Investigation, Formal analysis, Data curation, Conceptualization. **Philip Naden:** Writing – review & editing. **Subash Thapa:** Writing – review & editing. **Peter Gibbs:** Writing – review & editing.

## Declaration of competing interest

The authors declare no competing interests.

## Data Availability

No data was used for the research described in the article.
